# Supplementary material for: Evaluation the validity and reliability of the perceived medical school stress scale in Turkish medical students
Source: PLoS One. 2023 Aug 11;18(8):e0288769. doi: 10.1371/journal.pone.0288769 (PMC10420339; doi:10.1371/journal.pone.0288769)
Supplement: Attachment — Submitted filename: Reviewer # 4.docx [file pone.0288769.s003.docx]

Dear Reviewer,

We would like to thank you for your insightful comments and suggestions. We made all possible changes that were suggested and detailed the changes in the table below. Prior to responding to your comments, we want to inform you that all the revisions and improvements are highlighted red in the revised version of our manuscript. We sincerely appreciate your comments on our manuscript. We would like to thank you again for your valuable time and insight to strengthen our paper.

Yours truly,

Corresponding author on behalf of the authors.

|  | **Reviewer #4 Comment** | **Response** |
| --- | --- | --- |
| 1. | Maximum number of both scales should be added to the abstract. | We have added more information about PMSS and GAD-7 to the abstract section. |
| 2. | 'Medical Faculty' is not an appropriate key-word. | We have removed 'Medical Faculty' from keywords. |
| 3. | What is 'XXX' in line 94? | XXX was written for blinding. All changed to “Atatürk”. |
| 4. | A " is missed in line 135. | Corrected. |
| 5. | Psychometric properties of the original version should be added. | We have added psychometric properties and internal consistency of the original version. |
| 6. | The manuscript needs exact proof reading for any typo like '(24]' | The entire manuscript has been revised for mistypos, if any |
| 7. | What is definition of 'Income status'? | Students were asked about their own perceptions of their economic situation. They were asked to choose one of the definitions as good, medium or bad. |
