# Supplementary material for: Evaluation the validity and reliability of the perceived medical school stress scale in Turkish medical students
Source: PLoS One. 2023 Aug 11;18(8):e0288769. doi: 10.1371/journal.pone.0288769 (PMC10420339; doi:10.1371/journal.pone.0288769)
Supplement: Attachment — Submitted filename: Respond to Reviewer 1.-revision 2 docx.docx [file pone.0288769.s004.docx]

Dear Reviewers,

We would like to thank you for your insightful comments and suggestions. We made all possible changes that were suggested and detailed the changes in the table below. Prior to response your comments, we want to inform you that all the revisions and improvements are highlighted red in the revised version of our manuscript. We sincerely appreciate your comments on our manuscript. We would like to thank you again for your valuable time and insight to strengthen our paper.

Yours truly,

Corresponding author on behalf of the authors.

|  | **Reviewer 1 Comment**  I Thank you for considering the comments in revised article. Just for imprving manuscript I suggest that, | **Response to Reviewer 1 comment** |
| --- | --- | --- |
| 1. | using "Evauation" instesd of "Assessing" in topic. | We changed “assessing” as “evalaution” in the title as you recommend. |
| 2. | Table 2 are very confusing. unfortunatelly I couldnt understand what information have presented in this table (Test-re-test reliabilty or ICC). | Table 2 shows test-retest reliability.  We didn’t calculate test-retest reliability and ICC for pilot application.  For this reason, we seperated the ICC from pilot application section and it has been added to after convergent validity as you suggest. |
